# Supplementary material for: The Impact of the COVID-19 Pandemic on School-Aged Children with Fragile X Syndrome
Source: Genes (Basel). 2022 Sep 17;13(9):1666. doi: 10.3390/genes13091666 (PMC9498475; doi:10.3390/genes13091666)
Supplement: Supplementary file 1 [file genes-13-01666-s001.zip › genes-1892743-supplementary.pdf]

Supplementary Material
Full Study Survey

A) Demographics of the person with FXS

1. What is the birth sex of the person with FXS?
   - Male
   - Female
   - Prefer not to answer

2. How old is the person with FXS (in years)?

3. Which race/ethnicity best describes the person?
   - White
   - Hispanic or Latino
   - Black or African American
   - Native American or American Indian
   - Asian or Pacific Islander
   - Other: [fill in the blank]
   - Prefer not to answer

4. Which of the following best describes the region in which the person permanently resides?
   - Urban or metropolitan
   - Suburban
   - Rural
   - Other: [fill in the blank]
   - Prefer not to answer

5. What is your average annual household income?
   - Less than $50,000
   - $50-100,000
   - $100-150,000

- $150-200,000
- More than $200,000
- Prefer not to answer

6. How challenging is the **current** environment for the person with FXS compared to before the pandemic (around **February 2020**)?
    - Much more challenging
    - More challenging
    - About the same
    - Less challenging
    - Much less challenging

B) School Settings:

1. What best describes the person's educational setting in **February 2020** (pre-COVID)?
    - School for children with special needs (emotional, developmental, or behavioral problems- public or private)
    - Self-contained classroom in a regular school with no time spent in typical classroom
    - Self-contained classroom with some time spent with typical peers
    - Typical classroom with classroom support (pull out or in class; 1-to-1 aide; Collaborative Team Teaching (CTT) or Integrated Co-Teaching (ICT) classroom)
    - Typical classroom without support
    - Homeschooling
    - Online/ cyber schooling
    - No schooling, therapy only
    - No schooling or therapy
    - Other: [fill in the blank]
    - Don't know
    - Not applicable

2. What best describes the person's educational setting **now** (COVID-circumstances)?

- School for children with special needs (emotional, developmental, or behavioral problems- public or private)
- Self-contained classroom in a regular school with no time spent in typical classroom
- Self-contained classroom with some time spent with typical peers
- Typical classroom with classroom support (pull out or in class; 1-to-1 aide; Collaborative Team Teaching (CTT) or Integrated Co-Teaching (ICT) classroom)
- Typical classroom without support
- Homeschooling
- Online/ cyber schooling
- No schooling, therapy only
- No schooling or therapy
- Other: [fill in the blank]
- Don’t know
- Not applicable

3. What method of teaching is the person with FXS’s school using for the majority of the fall (**September-December 2020**)?
   - In person
   - Hybrid of online and in person learning
   - Fully online learning
   - Homeschooling
   - Other [fill in the blank]
   - No school

4. From **March** to **Now** (ongoing COVID pandemic), do you think the person’s overall academic performance (reading, writing, math) has:
   - Significantly increased
   - Mildly increased
   - Stayed the same
   - Mildly decreased
   - Significantly decreased

C) Therapy/ Services

1. Does the person receive any of the following services **through school** and how satisfied are you with the current services? (check all that apply, only received **through school**)

Scale for satisfaction: 1- very unsatisfied 2- unsatisfied 3- neutral 4- satisfied 5- very satisfied

Service	Frequency in **February 2020** or pre-COVID (hrs/week)	Was this service in person, online, or hybrid **pre-COVID**?	Frequency **now** (hrs/week)	Is this service in person, online, or hybrid **now**?	What is your current level of satisfaction? (using scale above)
Psychological/ Behavioral (including Applied Behavior Analysis)					
Social Skills Training					
Speech and Language Therapy					
Occupational Therapy					
Physical Therapy					
Sensory Integration Therapy					
Vocational Training					
Tutoring					
Counseling (including family support therapy)					
Other: [fill in the blank]					
None					

2. Does the person receive any of the following services **outside of school/ from a private setting** and how satisfied are you with the current services? (check all that apply, only received **outside of school/private setting**)

Scale for satisfaction: 1- very unsatisfied 2- unsatisfied 3- neutral 4- satisfied 5- very satisfied

Service	Frequency in **February 2020** or pre-COVID (hrs/week)	Was this service in person, online, or hybrid **pre-COVID**?	Frequency **now** (hrs/week)	Is this service in person, online, or hybrid **now**?	What is your current level of satisfaction? (using scale above)
Psychological/ Behavioral (including Applied Behavior Analysis)					
Social Skills Training					
Speech and Language Therapy					
Occupational Therapy					
Physical Therapy					
Sensory Integration Therapy					
Vocational Training					
Tutoring					
Counseling (including family support therapy)					
Other: [fill in the blank]					
None					

3. If the person with FXS's services have transitioned to an online format, how do you think the person is adjusting to this change?
   - Adjusting very well
   - Adjusting well
   - Adjusting acceptably

- Adjusting poorly
- Adjusting very poorly
- Not applicable

4. Which services or therapies do you think have been the most beneficial to the person with FXS over the course of the pandemic?

Matrix: 1-very unhelpful 2-mildly unhelpful 3-neutral 4-mildly beneficial 5-very beneficial

- Psychological/ Behavioral (including Applied Behavior Analysis)
- Social Skills Training
- Speech and Language Therapy
- Occupational Therapy
- Physical Therapy
- Sensory Integration Therapy
- Vocational Training
- Tutoring
- Counseling (including family support therapy)
- Other: [fill in the blank]

D) Behavior:

1. Did the person have any of the following issues before the pandemic (around **February 2020**)?

   Matrix: 0 = no, 1 = unsure, 2 = mild, 3 = moderate, 4 = severe

- Attention problems (trouble focusing, constantly switching tasks, ADD)
- Hyperactivity (restlessness, fidgeting, noncompliance)
- Aggressive behavior (hitting, biting, verbal hostility)
- Irritability (agitation, crying)
- Perseverative behavior (repetitive speech, need to do things over and over again)
- Self-injury (hitting self, biting self, banging head on objects)
- Anxiety (phobias, panic attacks, OCD)
- Depression (withdrawal from tasks, extended periods of sadness, lethargy)
- Hypersensitivity/ overreaction to stimuli (sensory issues)

- Sleep disorders (insomnia, sleep apnea)
- Other [fill in the blank]
- None

2. Please indicate any behavioral changes you have observed in the person with FXS during the pandemic (between **February 2020** and **now**).

	Very much improved	Much improved	Minimally improved	No change	Minimally worse	Much worse	Very much worse	Not applicable
Attention Problems								
Hyperactivity								
Aggressive behavior								
Irritability								
Perseverative behavior								
Self-injury								
Anxiety								
Depression								
Hypersensitivity/ overreaction to stimuli								
Sleep disorders								
Other								
None								

E) Medication:

Medication includes only prescribed medications.

1. Is the person taking more medication **now** than he/she was taking before the pandemic (around **February 2020**)?
   - No
   - No more medications but the dosages were increased
   - Yes, 1 more medication
   - Yes, 2 more medications
   - Yes, 3+ more medications
   - Not applicable: [please explain]

2. What were the causes of these changes in the medication? (select all that apply)
   - Natural course of changes as planned before or not impacted by COVID-19
   - The COVID-19 pandemic impact on behavior
   - Overall health shifts
   - Other [fill in the blank]
   - I don't know
   - Not applicable

F) Health Visits

1. Has the person had a doctor's appointment with a FXS specialists during the pandemic (since **February 2020**)?
   - No
   - Yes, both in person and virtual
   - Yes, only virtual
   - Yes, only in person

2. How likely is it that you will go to an **in-person** appointment in the following situations?

Matrix: 1- very unlikely 2- unlikely 3- neutral 4- likely 5- very likely

   - Behavioral changes
   - Medical advice

- Regular follow-up
- Annual Physical
- Vaccines
- Paperwork
- Research participation
- Exposure to personnel and protective wear (mask)
- Other: [fill in the blank]

3. How likely is it that you will go to a **virtual** appointment in the following situations?

Matrix: 1- very unlikely 2- unlikely 3- neutral 4- likely 5- very likely

- Behavioral changes
- Medical advice
- Regular follow-up
- Annual Physical
- Vaccines
- Paperwork
- Research participation
- Exposure to personnel and protective wear (mask)
- Other: [fill in the blank]

G) Daily Living Skills

1. During the ongoing pandemic period (between **February 2020** and **now**), how do you think the person's overall performance of activities of daily living (feeding, bathing, dressing, toileting, speaking in public, social activities, driving, managing money) has changed?

- Significantly increased
- Mildly increased
- Stayed the same
- Mildly decreased
- Significantly decreased

2. Were the person's daily living skills affected by the COVID pandemic (between **February 2020** and **now**)?

	Very much improved	Much improved	Minimally improved	No change	Minimally worse	Much worse	Very much worse	Not applicable
Feeding								
Bathing								
Dressing								
Toileting								
Social skills (including in person communication)								
Managing money								
Driving								
Other [fill in the blank]								

H) Mask Use

1. How would you describe the person's ability to wear a mask when asked or required?
   - Excellent: no challenges
   - Good: wears a mask a majority of the time
   - Acceptable: wears a mask sometimes
   - Poor: wears a mask minimally
   - Very poor: never wears a mask

2. Which of these describe the challenges the person has with their masks if any? (Please check all that apply).
   - Sensory: Sensory issues prevent wearing a mask
   - Understanding: Do not understand the necessity of a mask
   - Change in routine: Because it is novel they have to get used to the idea of a mask
   - Behavioral: Increases maladaptive behaviors
   - Other (please explain)
   - I don’t know
   - Not applicable

I) Free response:

1. Please use this space to expand upon any additional notes you would like to include regarding school, services, behavior, medication, health visits, daily living skills, or masks, that you feel were not covered in the above multiple choice questions.

2. What ideas do you have that you think would help the person during this or any pandemic (e.g. daily schedules, activities to do during the day, strategies for keeping anxiety down)? Please be as specific as you can.
